# Spatio-temporal spillover risk of yellow fever in Brazil

**DOI:** 10.1186/s13071-018-3063-6

**Published:** 2018-08-29

**Authors:** RajReni B. Kaul, Michelle V. Evans, Courtney C. Murdock, John M. Drake

**Affiliations:** 1 Center for the Ecology of Infectious Diseases, University of Georgia, Athens, GA USA; 2 Odum School of Ecology, University of Georgia, Athens, GA USA; 3 Department of Infectious Diseases, University of Georgia, Athens, GA USA; 4 Center for Tropical and Global Emerging Diseases, University of Georgia, Athens, GA USA; 5 Center for Vaccines and Immunology, University of Georgia, Athens, GA USA; 6 River Basin Center, University of Georgia, Athens, GA USA

**Keywords:** Yellow fever, Risk mapping, Vectors, Brazil, Spatial epidemiology, Arboviruses

## Abstract

**Background:**

Yellow fever virus is a mosquito-borne flavivirus that persists in an enzoonotic cycle in non-human primates (NHPs) in Brazil, causing disease in humans through spillover events. Yellow fever (YF) re-emerged in the early 2000s, spreading from the Amazon River basin towards the previously considered low-risk, southeastern region of the country. Previous methods mapping YF spillover risk do not incorporate the temporal dynamics and ecological context of the disease, and are therefore unable to predict seasonality in spatial risk across Brazil. We present the results of a bagged logistic regression predicting the propensity for YF spillover per municipality (administrative sub-district) in Brazil from environmental and demographic covariates aggregated by month. Ecological context was incorporated by creating National and Regional models of spillover dynamics, where the Regional model consisted of two separate models determined by the regions’ NHP reservoir species richness (high *vs* low).

**Results:**

Of the 5560 municipalities, 82 reported YF cases from 2001 to 2013. Model accuracy was high for the National and low reservoir richness (LRR) models (AUC = 0.80), while the high reservoir richness (HRR) model accuracy was lower (AUC = 0.63). The National model predicted consistently high spillover risk in the Amazon, while the Regional model predicted strong seasonality in spillover risk. Within the Regional model, seasonality of spillover risk in the HRR region was asynchronous to the LRR region. However, the observed seasonality of spillover risk in the LRR Regional model mirrored the national model predictions.

**Conclusions:**

The predicted risk of YF spillover varies with space and time. Seasonal trends differ between regions indicating, at times, spillover risk can be higher in the urban coastal regions than the Amazon River basin which is counterintuitive based on current YF risk maps. Understanding the spatio-temporal patterns of YF spillover risk could better inform allocation of public health services.

**Electronic supplementary material:**

The online version of this article (10.1186/s13071-018-3063-6) contains supplementary material, which is available to authorized users.

## Background

Yellow fever virus (YFV), a mosquito-borne flavivirus, began re-emerging in Brazil in the early 2000s, gradually spreading from the Amazon River basin towards the southeastern region of the country [1, 2]. Unlike other mosquito-borne diseases in Brazil, such as malaria and dengue, YFV circulates in a sylvatic transmission cycle among non-human primate (NHP) reservoirs maintained by *Haemagogus* and *Sabethes* mosquitoes, with spillover occurring when mosquitoes transmit outside this cycle to human hosts [3, 4]. Thus, YFV spillover events leading to human yellow fever (YF) disease are closely tied to population dynamics and community composition of both mosquitoes and NHP. Beginning in late 2016, Brazil experienced a YF outbreak of over 450 cases, more cases than had been reported in the previous fifteen years combined [5]. The majority of these cases were outside the previously defined range of YF and near densely populated, urban areas. To date, the Brazilian Ministry of Health classifies these cases as “sylvatic”, or the direct result of a spillover event due to transmission of YFV from local NHP reservoirs to humans *via Haemagogus* and *Sabethes* mosquitoes. The unexpected emergence of YF in these regions previously considered low-risk highlights the need for a better understanding of the spatial and temporal patterns of YF spillover risk.

Infectious disease mapping is one such method to predict spatial patterns in disease transmission risk [6]. Predictive maps of disease risk use statistical methods to account for disease transmission dynamics *via* their relationship with environmental and socio-demographic covariates. This approach has been applied to a range of infectious diseases, including Ebola [7, 8], malaria [9, 10] and dengue [11, 12]. Past efforts at mapping YF have focused primarily on environmental variables as drivers of YF spillover dynamics [13–15]. However, these studies have relied on static environmental variables, ignoring multi-year and annual cycles in environmental conditions and spillover events which occur on seven and fourteen year cycles [16, 17]. Multi-year cycles are due to the El Niño-Southern Oscillation [18] and the accumulation of susceptible hosts in NHP populations [16]. Spillover events are also seasonal, with the majority of cases occurring during the rainy season (approximately December-April), when mosquito vector abundances are highest [19]. Predictive models that focus on a snapshot in time, or a normalized average of climate data, are therefore unable to capture temporal changes in spillover risk.

Because the compositions of mosquito and NHP communities vary over space, it might be that the processes and corresponding environmental drivers of YF spillover are dependent on the underlying ecological context. For example, in the West Nile virus (WNV) system in the USA, WNV is positively associated with urban land cover in the northeast and with agricultural land cover in the west, due to different habitat preferences of vectors of the disease, which differ across regions [20]. Like the USA, Brazil contains multiple biomes, with Amazon rainforest in the northwestern region, cerrado (savanna) in the central region, and Atlantic rainforest in coastal areas in the southeast. We reasoned that the processes and corresponding environmental drivers of YF spillover are dependent on the underlying ecological context or region. Multiple models, unlike a single model, can incorporate different directions and magnitudes of relationships between environmental variables and YF spillover. By assuming a single homogeneous process of spillover across the country, current models of spatial risk of YF spillover do not allow for the existence of a variety of spillover processes, and therefore cannot identify their corresponding environmental drivers.

Past spatial mapping of YF risk in Brazil has assumed a temporally constant risk or similar environmental drivers of YF spillover across all regions of Brazil, regardless of their NHP species richness [14, 15, 21, 22]. Here, we first present a statistical model (National model) that incorporates monthly variation in covariates to predict the propensity of YF spillover across municipalities (sub-state administrative units) of Brazil. Secondly, we better contextualize the spillover process by fitting models to two contiguous regions determined by a natural break in NHP reservoir species richness. The composite of these models are presented as the Regional model. Finally, we present the models’ predictions of the spatio-temporal risk of YF transmission by month across Brazil from 2001 to 2013 for the National and Regional models.

## Methods

**Fig. 1:**
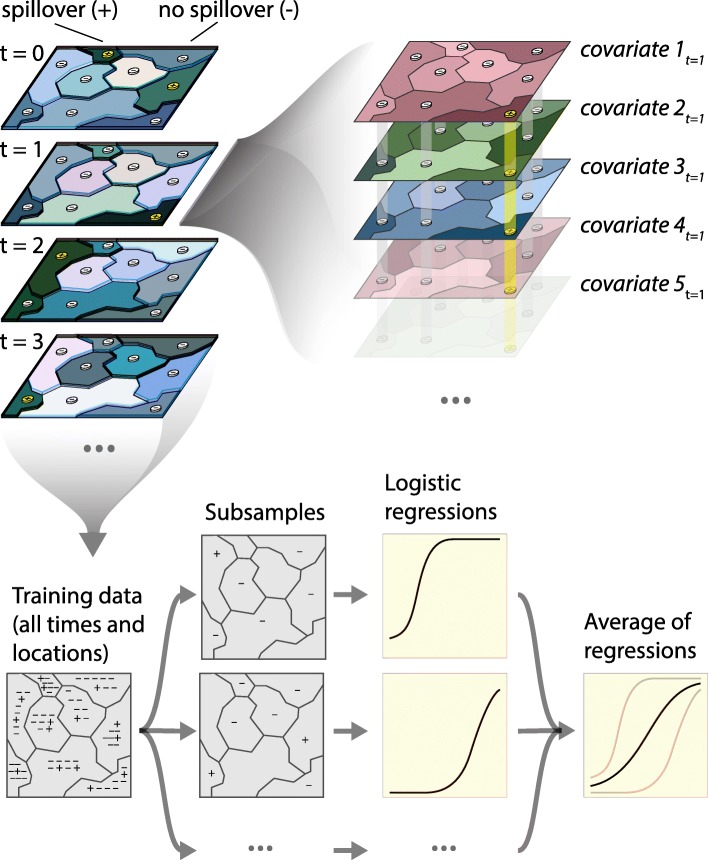
Conceptual diagram of modeling methods. The dataset was aggregated by month and municipality (top panel) before being split into training (70%) and withheld testing (30%) datasets. Models were fit to 500 data subsamples, which consisted of 10 spillover events and 100 background observations (lower panel). The bagged logistic model predictions are the average of subsampled dataset models. Spatial dependence was not considered in the model

YF spillover propensity was estimated for each month between January 2001 and December 2013 using 12 environmental and socio-demographic covariates for each municipality by a bagged logistic regression model (Fig. 1, Table 1). The spatial and temporal unit of the model, municipality and month (hereby referred to as municipality-month) was based on the finest resolution of epidemiological data reported by the Brazilian Ministry of Health. Municipality-months with at least a single reported YFV case were given a positive label for the binary response variable, spillover event. Data collection methods are briefly reported here, with additional details reported in Additional file 1.

**Table 1:** Summary of data sources used in the model (see Additional file 1 for additional information on variable collection)

Data type	Temporal resolution	Raw spatial resolution	Source	Extreme variable?
Yellow fever incidence	Monthly	Municipality	MS	–
Population density	Yearly	Municipality	MS	–
Land surface temperature	Monthly	0.05°	LPDAAC	Yes
Normalized difference vegetation index	Monthly	1 km	LPDAAC	Yes
Average hourly rainfall^a^	Monthly	0.25°	TRMM	Yes
Fire density^a^	Monthly	1 km	FIRMS	Yes
Non-human primate species richness^a^	Static	Municipality	IUCN	–
Agricultural and non-human-primate overlap^a^	Yearly	1 km	IUCN/ LPDAAC	–
Maximum probability of mosquito vector occurrence	Static	0.04167°	VectorMap	–

*Abbreviations*: *MS* Brazilian Ministry of Health, *LPDAAC* NASA Land Processes Distributed Active Archive Center, *TRMM* Tropical Rainfall Monitoring Mission, *FIRMS* Fire Information for Resource Management System, *IUCN* International Union for Conservation of Nature

^a^Variable was cube root transformed prior to model construction

### Data collection

We chose environmental and socio-demographic covariates that are suspected drivers of YF disease transmission by either modifying the host population, mosquito population or host proximity to competent non-human primate (NHP) reservoirs (Table 1). Variables were downloaded at the monthly, yearly, or static temporal resolution, as determined by the availability of data for the whole country of Brazil. In the case where annual values were the smallest temporal resolution, values were held constant for the year. Variables were spatially averaged by area to the resolution of the municipality to provide a mean value for each municipality-month. Aggregating spatial variables can lead to bias (e.g. the modifiable areal unit problem) by analyzing the relationship between variables at a coarser spatial scale than the process of spillover occurs [23, 24]. However, we limited bias introduced by aggregation by conducting our analysis at the finest grain possible given the availability of epidemiological data. Further, we believe conducting an analysis at the scale of the municipality rather than pixel-level is more relevant to public health decision-making as this corresponds to the smallest administrative level. All variables were visually inspected for normality and cube root transformed if needed, except for population density, which was log_10_-transformed (Table 1).

Mosquitoes are extremely sensitive to changes in temperature and relative humidity, and their abundance is often correlated with rainfall [12, 25, 26]. We therefore chose mean monthly temperature (hereby referred to as temperature), the Normalized Difference Vegetation Index (NDVI), and mean hourly rainfall averaged over a month (hereby referred to as rainfall) as variables relevant to mosquito population dynamics. While daily temperature variability and extremes are important for mosquito dynamics [27, 28], environmental variables are not available at this fine of a temporal-scale for the entire country of Brazil. We therefore used monthly averages to represent the climatic profile of a municipality corresponding to the temporal resolution of the epidemiological reports. Preliminary data analysis revealed spatial minima, maxima and mean variables to be highly correlated (*ρ* > 0.90, Additional file 1: Figure S1). Univariate analyses on the training data found no difference in explanatory power amongst variables (Additional file 1: Table S1). Therefore, we chose the spatial mean to represent each environmental variable to reduce bias due to collinearity [29]. In addition to environmental variables that can influence mosquito abundances, we also included the species ranges of mosquito vectors of YF in our model. We estimated the occurrence of mosquito vectors from published MaxEnt models of distributions of three YF vector species (*Hg. leucocelaenus*, *Hg. janthinomys* and *Sa. chloropterus*). The maximum probability amongst the three species was chosen for each municipality, resulting in the maximum probability of a mosquito vector occurring within that municipality.

Transmission from the sylvatic cycle to human hosts occurs when human hosts are in proximity to NHP reservoirs of the pathogen. Human proximity to NHPs was captured in three variables. First, we created a variable of fire density, as a representation of anthropogenic land conversion [30]. Secondly, we calculated the species richness of NHPs susceptible to YF per municipality to represent reservoir abundance. In general, host species richness positively correlates with spillover events of diseases of zoonotic origin [31]. Other factors, such as species’ competences, relative abundances, and overall community structure, can further influence spillover risk [32, 33]. However, the role of individual species in YF transmission and the population abundances of NHPs across all of Brazil is not known. Finally, as a proxy for longer-term NHP-human contact, we calculated the proportion of a municipality’s land area that is both agricultural land use and within a primate species’ range. To calculate this proportion of agricultural land overlapping NHP species’ ranges, we overlaid species range maps with land cover maps of agricultural land use. We then calculated the proportion of total municipality area that was both in agricultural use and within a genus range and summed across all nine genera, resulting in a value of 0–9 per municipality per year.

Additional variables were constructed to capture extreme events, which are considered to be triggers for socio-ecological events like YF spillovers. The three environmental covariates intended to reflect mosquito dynamics (temperature, NDVI and rainfall) along with fire density were scaled to the maximum value for that specific calendar month and municipality. The inclusion of anomalies allowed us to distinguish between seasonal trends and particularly extreme events acting as triggers [7].

Human host population dynamics were captured by annual estimates of human population by municipality, obtained from the Brazilian Institute of Geography and Statistics. This was converted to population density based on municipality area, and then log_10_-transformed. Higher frequency population density estimates or proportion of the population vaccinated were not available by municipality.

Finally, a spillover occurred in a municipality-month that had one or more confirmed YF cases. We used the monthly confirmed cases of YF reported by the Brazilian Ministry of Health Notification of Injury Information System (Sianan Net) to determine whether a spillover event occurred for each municipality in each of the 156 months between January 2001 and December 2013. Exploratory analysis indicated the majority of YF cases were single cases per municipality-month (mean 1.83 ± 2.46 YF cases per municipality-month, Additional file 1: Figure S2). Given the consistent reporting of single cases per municipality-month, we collapsed the continuous case counts data into the binary spillover variable. In municipality-months with multiple cases reported, the binary classification assumes that at least one case is the result of sylvatic transmission. Municipality-months with a single case and those with multiple cases are equally weighted as a single spillover event.

The compiled dataset had 867,360 observations (5560 municipalities times 156 months), 106 of which were positive for a spillover event (Table 2). No additional variable selection was conducted before model training and testing.

**Table 2:** Dataset summary. Training and testing dataset used to build the National model, which was then subset into the low reservoir richness (LRR), and high reservoir richness (HRR) Regional models

Model	Training dataset		Testing dataset		Whole dataset	
	Positive	Background	Positive	Background	Positive	Background
National	74	607,077	32	260,177	106	867,254
LRR	59	584,263	27	250,251	86	834,514
HRR	15	22,814	5	9926	20	32,740

### Predictive model

We used a bagged logistic regression to predict the monthly propensity of YF spillover per municipality across Brazil, which we refer to as the “National model” (Fig. 1). Bagging (bootstrap aggregating) is an ensemble machine learning approach that combines the predictive power of many less informative models to provide an overall more accurate prediction [34, 35]. The less informative models are constructed from random small subsets of the full dataset. The final model consists of an average of the less informative models. Bagging is also particularly robust with noisy or sparse data, ensuring models are not over fit to the relatively few positive observations [34].

We used a balanced spatial and temporal sampling design *via* the *BalancedSampling* package in R to split the data into training (70%) and testing (30%) sets, preserving the proportion of municipality-months with a spillover event (positive observations) in each [36]. This sampling design ensured there was no spatial or temporal trend in our training and testing data, which is particularly important given the severe imbalance of positive to negative or background observations (positive observations are ≈0.01% of the dataset) [37]. The training data were used to fit 500 logistic regression models with main effects of the 12 variables. Each logistic regression model was fit to a randomly sampled without replacement subsample of data consisting of 10 and 100 positive and background points in the training dataset, respectively. The final bagged logistic regression model is the average of the logistic regressions.

**Fig. 2:**
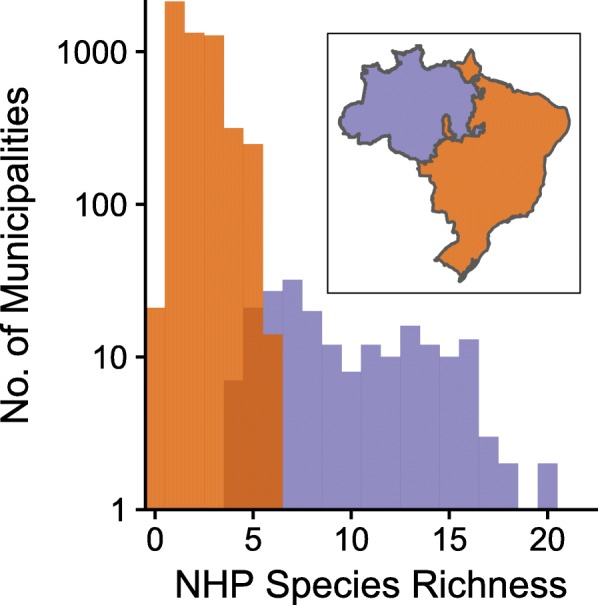
Distribution of NHP species richness by municipality. Plot of distribution of non-human primate species richness per municipality, colored by the break used to determine areas of high reservoir richness (purple) and low reservoir richness (orange). Inset is a map of the two regions

The data used for the national model above were further divided into two regional datasets, based on species richness of non-human primate reservoirs. These datasets were used to build separate high reservoir richness (HRR, > 5 NHP reservoir species) or low reservoir richness (LRR, ≤ 5 NHP reservoir species) models, both of which still included NHP richness as a covariate. We refer to the combination of these models as the “Regional model”. This division was determined by a natural break in the distribution of the species richness data, which generally corresponded with a geographic break (Fig. 2). In a few instances, municipalities with a low reservoir richness (≤ 5 NHP reservoir species) were included in the HRR region, and vice versa to create two contiguous regions. This data driven delineation of regions within the Regional model allowed for potentially different relationships between environmental covariates and spillover risk in the HRR and LRR regions while preserving the maximum number of positive observations within the datasets.

We calculated the performance of each model *via* the area under the receiver-operator curve (AUC) of the withheld testing data. The AUC represents the model’s classification performance and can range from 0.0 to 1.0, where a value of 0.5 is the equivalent of random guessing while an AUC of 1.0 indicates a perfect prediction [38]. Relative variable importance was assessed by the difference in median AUC of 100 permutations to the AUC of the original model, scaled to the largest decrease in AUC due to permutation of a single variable.

## Results

Eighty-two of 5560 municipalities reported YF cases from 2001 to 2013. The number of repeated spillover events in a single municipality was low. Roughly 77% (63) of municipalities reported cases in only a single month, 18% (15) reported cases in two months during the 13 year period. Only 3 municipalities reported cases in 3 months, and a single municipality reported cases in four months. Municipalities with more than 2 spillover events were all located Minas Gerais, a southeastern state with locally-acquired cases in the 2017 and 2018 outbreaks.

Model predictive accuracy was high for all three datasets. The AUC values for models evaluated on the training dataset were 0.81, 0.79 and 0.88 for the national dataset, LRR and HRR regions, respectively. When evaluated on the testing dataset, the AUC was 0.80 for both the National and LRR Regional models, while the predictions on the HRR testing dataset had an AUC of 0.63.

**Fig. 3:**
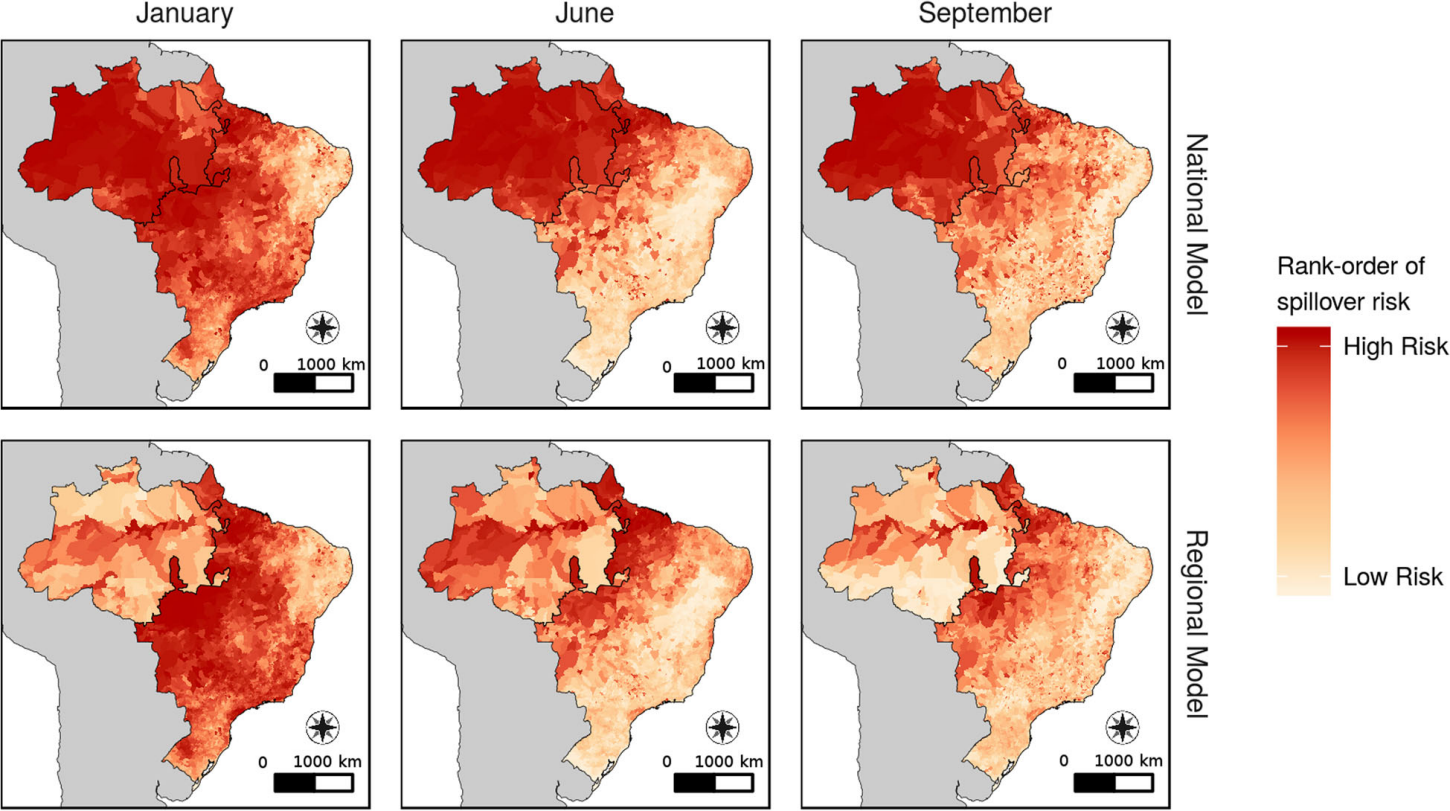
Predicted spatial risk of yellow fever spillover. Propensity of yellow fever spillover in January, June, and September of 2008. Raw outputs of the model for each municipality-month are rank-ordered to allow for comparison across models. Results from the National model are on the top row and the Regional model are on the bottom row. Black outline represents the split between HRR (northwest) and LRR (southeast) regions. The outline in the national model is for reference only. See supplemental video for entire time series. Map projection: SAD69 Brazil Polyconic. Data source: 2001 municipality boundaries, Brazilian Institute of Geography and Statistics

**Fig. 4:**
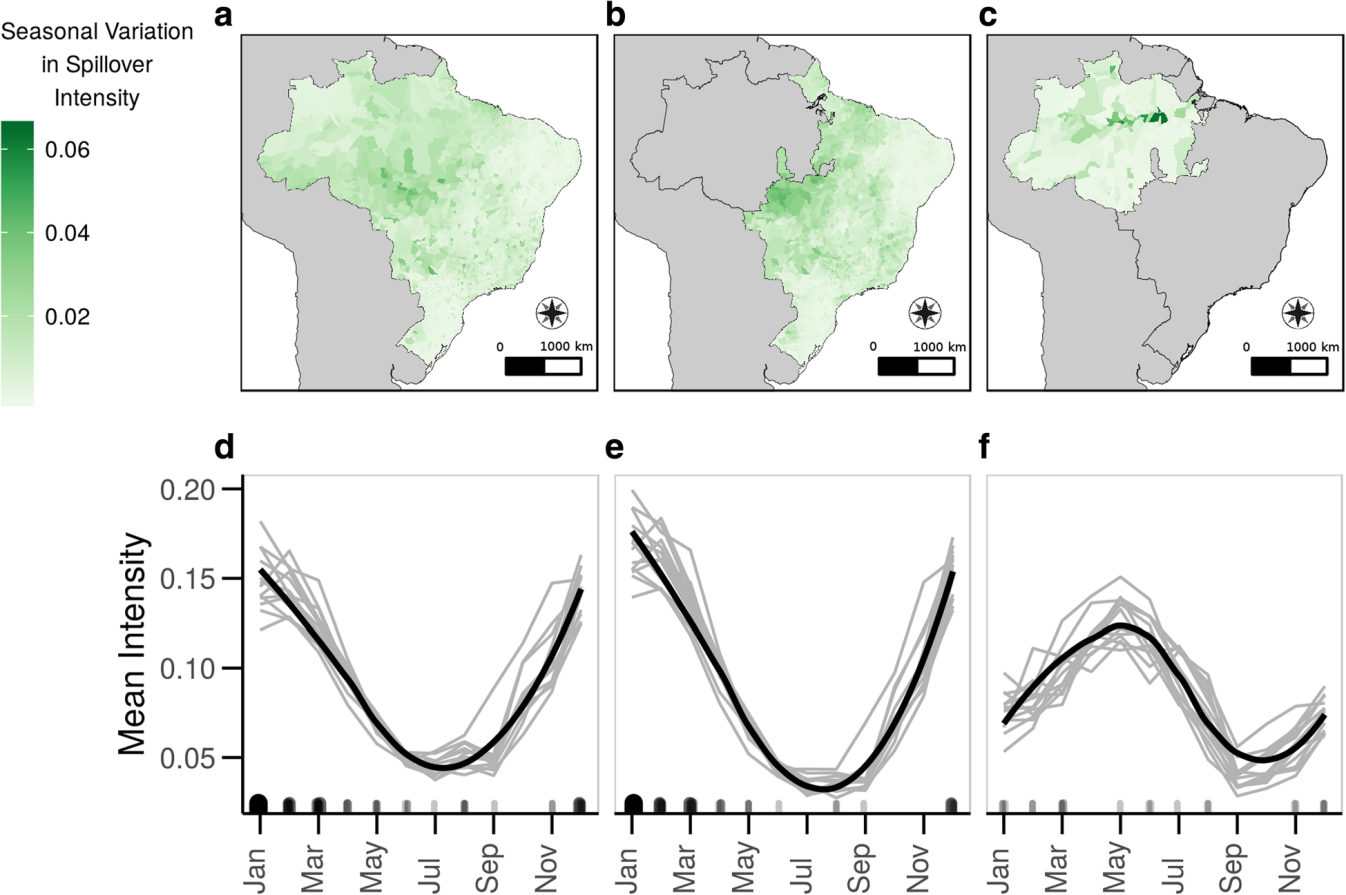
Variation of yellow fever spillover intensity in space and time. Plots of variance of the predicted spillover intensity throughout the 13-year time series from the National model (**a**), low reservoir richness Regional model (**b**), and high reservoir richness Regional model (**c**). Darker municipalities are predicted to have greater seasonality in spillover risk than lighter municipalities. The seasonal pattern in model predictions are shown by monthly averages of predicted spillover intensity across the entire study area of Brazil for the National model (**d**), within the low reservoir richness Regional model (**e**), and within high reservoir richness Regional model (**f**). Gray lines represent an individual year of data with overall mean in black. Rug along x-axis represents true spillover events, with larger and darker shapes representing more spillover events during that calendar month. Map projection: SAD69 Brazil Polyconic. Data source: 2001 municipality boundaries, Brazilian Institute of Geography and Statistics

The predicted risk of YF spillover varied by season, generally peaking in January for the National and LRR Regional models (Fig. 3). The temporal risk in the HRR model was asynchronous to the other two models, and peaked in May (Fig. 4f). A supplemental movie shows the predicted risk of YF spillover for the entire time series (Additional file 2). The municipalities along the Amazon River and surrounding São Luís, Rio de Janeiro and São Paulo had the greatest change in spillover risk throughout the year (Fig. 4a-c). This annual variation was more pronounced in the Regional models.

We conducted a slope test for each municipality from 2001 to 2013 to explore long-term trends in the predicted intensity of yellow fever spillover. The majority of municipalities saw no long-term trend in predicted intensity over the 156 months included in the dataset (National model: 5028 of 5560 municipalities; Regional model: 5045 of 5560 municipalities). More municipalities experienced a decrease in predicted spillover intensity (National: 489; Regional: 482) than experienced an increase in intensity (National: 43; Regional: 33). Intensity tended to increase over the study period in municipalities along the Amazon River and in the southeastern region of Brazil and decrease along the coast (Additional file 1: Figure S4). When considering calendar months individually for the Regional model, predicted spillover intensity increased the most in July and August between 2000 and 2013, particularly in the southeastern region of the country (Additional file 1: Figure S5), however these months still had the lowest spillover intensity over all. When averaging across municipalities, the highest predicted annual risk was in 2008, followed by 2001.

The area of highest risk differed by model. In the National model, the HRR region had high predicted values of YF spillover risk across all 156 months and the LRR region experienced seasonal shifts in spillover intensity. In contrast, the Regional model predicted high risk of spillover in municipalities near the Amazon River, with lower risk in the higher elevation areas near Brazil’s northern border. As in the National model, the LRR region in the Regional model had seasonal variability in the risk of YF spillover, with a higher risk of spillover from November to March.

**Fig. 5:**
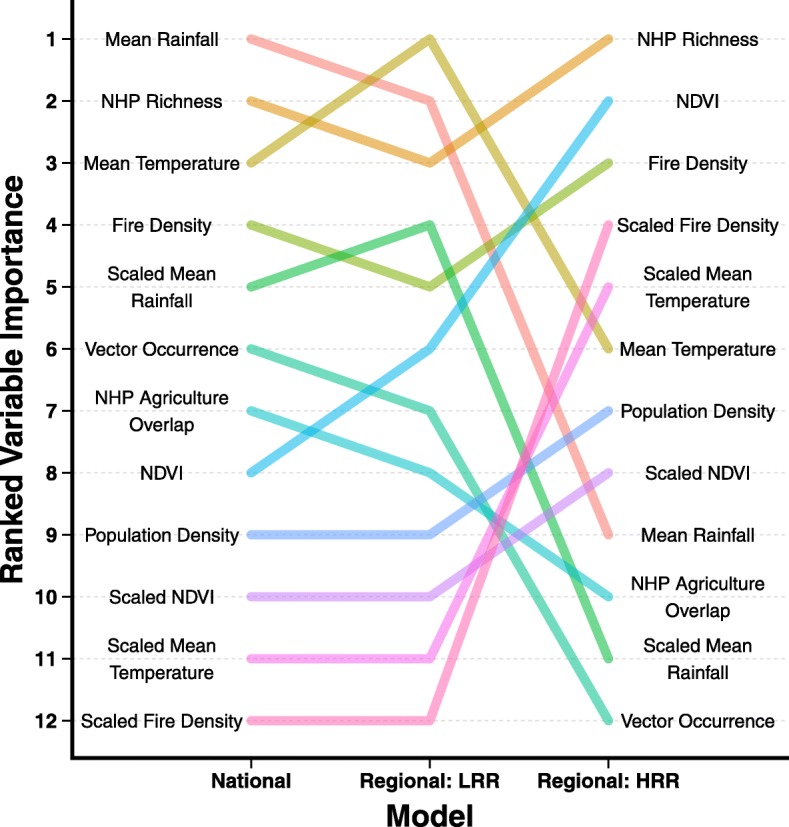
Rank order of median variable importance. The median variable importance was calculated for the National, low reservoir richness (LRR), and high reservoir richness (HRR) Regional models based on 100 permutations per variable within a model. The variables were ranked from most important (1) to least important (12) for model accuracy

The ranked order of variable importance differed between the models (Fig. 5). However, NHP richness was consistently highly ranked. The LRR Regional model and the National model had a similar rank order of variable importance, sharing the top three most important variables (rainfall, temperature, and NHP richness). This is perhaps expected given that the LRR dataset is 96% of the national dataset. Anomalous conditions as captured in the scaled covariates are of greater importance in the HRR Regional model than the other two models. However, the type of anomalous condition influences the covariates’ importance. Scaled rainfall is ranked in the top half of the ranked variables for the National and LRR Regional models, but is less important in the HRR Regional model. The scaled covariates of temperature and fire density follow the opposite pattern, increasing in importance for the LRR Regional model. To a lesser extent, the scaled NDVI follows the same pattern as the anomalous temperature and fire density covariates. The largest change in the rank order between the three models was rainfall (difference of 8), followed by scaled rainfall and scaled fire density (tied with a difference of 7), then NDVI and vector occurrence (tied with a difference of 6).

**Fig. 6:**
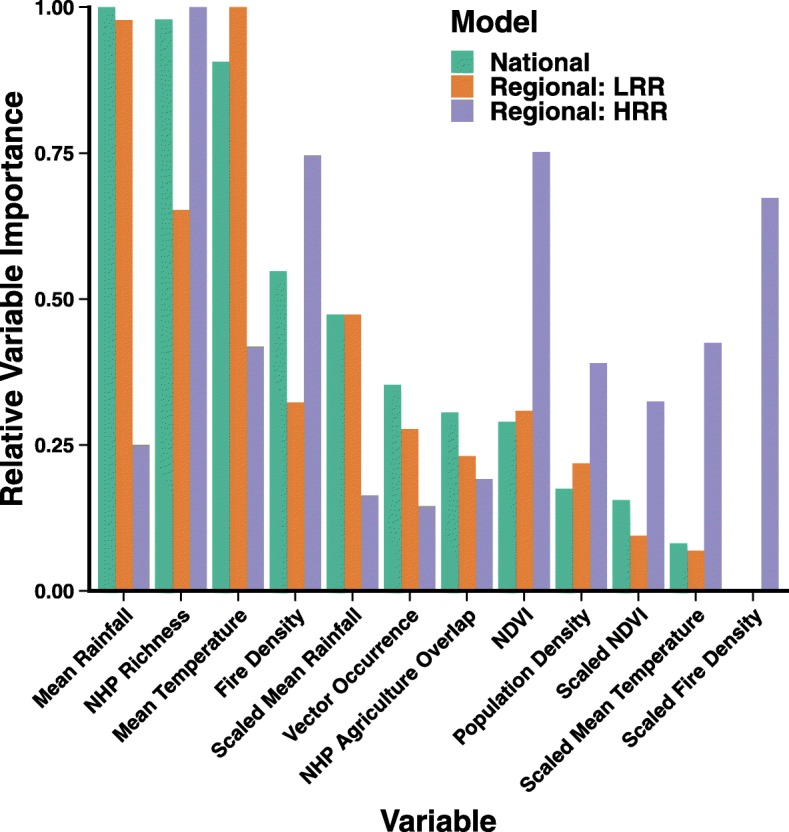
Variable importance for the National and Regional model. The median variable importance was calculated for the National, low reservoir richness (LRR), and high reservoir richness (HRR) Regional models based on 100 permutations per variable within a model. Values are the decline in AUC (∆AUC) due to permutation from the original model scaled to the largest ∆AUC within each model

The central tendencies of the relative variable importance, as measured by ∆AUC, for the three models included a natural break in the variables (Fig. 6). The top three variables in the National model (rainfall, NHP richness and temperature) were similar, while the first and second ranked variables for the LRR Regional model (temperature and rainfall) were very similar. In the HRR Regional model, the highest ranked variable (NHP richness) was substantially more important than the second through fourth variables (NDVI, fire density and scaled fire density), which were clustered. However, in general the ∆AUC for any variable permutation was small (less than 0.05 decrease from model AUC) indicating that model performance is not driven by a single covariate.

## Discussion

Yellow fever has re-emerged in Brazil, and is spreading into new regions. This range expansion is reflected in the most recent vaccine recommendation maps [14]. Accompanying this geographic expansion is an increase in the number of cases of YF, with large outbreaks in 2016/2017 and 2017/2018 (as of June 2018). Although a vaccine exists for the disease, understanding of the spatial and temporal risk of the spillover has been insufficient to predict where vaccines, a limited resource, should be distributed. We created retrospective time-varying risk maps of YF spillover based on environmental and demographic variables, identifying regions at risk of YF spillover throughout the year. In addition to the Amazon River basin, our maps highlight periods when the urban coastal region, a densely-populated area at the edge of YF expansion, is at an increased risk of disease spillover from NHP reservoirs. This approach can be expanded to incorporate near real-time data for risk mapping and decision support.

While most municipalities did not experience a long-term increase or decrease in intensity of spillover over the study period, those that did increase are in the southeastern and northern regions of Brazil. This agrees with other studies that have found YF virus to be spreading towards the southeast region in the past two decades, expanding from the enzoonotic zone to the transition zone [2, 39]. Our model found municipalities to have the highest predicted intensity of YF spillover in 2008, which corresponds to a large outbreak in Brazil’s southern states during that year [40]. Interestingly, we found the predicted intensity of a yellow fever spillover event to increase in July and August from 2001 to 2013, suggesting a lengthening of the season during which yellow fever is transmitted. However, this may also be an artefact of the time period used in our analysis, which ended with multiple consecutive La Niña years. La Niña years are characterized by a lengthening of the rainy season [41], during which vectors of YFV may be more abundant, leading to higher incidence of spillover into human populations. Regional climate cycles have been linked to transmission of vector-borne diseases elsewhere [29, 42, 43], and it is likely that changes in precipitation and temperature resulting from La Niña and El Niño events drive YF spillover in Brazil as well.

By modeling regional spillover risk separately in high and low NHP richness regions, we identified different seasonal patterns between regions. The largest difference in seasonal patterns between the National and Regional model is in the Amazon River basin, which shifts from a consistently high risk area in the National model (Fig. 4a) to alternating between high and low risk through the year in the Regional model (Fig. 4c). We found different patterns in the seasonal peaks of high spillover propensities when comparing between the LRR and HRR regions within the Regional model. This asynchrony of peak spillover propensity implies that at given times in the year, spillover risk can be higher in the urban, coastal regions than the Amazon River basin. This is contrary to contemporary maps of YF distribution in Brazil [14], but is consistent with the geographic distribution of the recent outbreaks of YF in 2016–2018.

There was a stark difference in the variables driving spillover in the National and LRR Regional models (which had a similar ranking of variable importance), and the HRR Regional model (Fig. 5). For the National and LRR Regional models, rainfall, temperature, and NHP species richness were the three most important variables. These variables were chosen a priori to represent vector and reservoir dynamics, and their ranking suggests that it is the combination of the presence of NHP reservoirs and high environmental suitability for mosquito vectors that leads to YF spillover. Other mosquito-borne diseases in South America are strongly correlated with climatic variables [38, 44, 45]. Similarly, we find evidence that YF spillover events are driven by temperature and rainfall in the LRR region. However, rainfall is less important in the HRR Regional model. Unlike the LRR region, the HRR region is composed entirely of Amazon rainforest, which receives over 4m of rainfall annually and has low potential evaporation rates [46], allowing standing water, which serves as mosquito habitat, to remain throughout the year. Although the onset of the rainy season in the Amazon can lead to an increase in the abundance of YFV vectors [47], this is dependent on species, and vectors of YF persist year round [48]. In the HRR region, therefore, it is likely that vector populations are not strongly limited by rainfall, supporting our results. This context-dependence of the effect of individual environmental variables on mosquito-borne disease has been shown for malaria [49] and should be further explored for the case of YF.

Further, landscape characteristics such as NDVI and fire density are more informative than climatic variables in the HRR region of the Regional model. NDVI is a measure of vegetative cover that, in Brazil, is a lagged response to global climate that represents seasonal climate conditions corresponding to the wet and dry season [50, 51]. In this way, it can be thought of as another climatic variable. In addition to climate, NDVI values are strongly influenced by land clearing techniques that rely on fire [51]. Given the relationship between NDVI and fire-fallow agriculture, and the importance of fire density in the HRR Regional model, human modification of the landscape *via* deforestation could be driving spillover events in this region. Malaria prevalence and the abundance of anopheline species often increases following human migration and conversion of land from forest for agricultural development in the Amazon [52, 53] and spillover of another vector-borne disease of NHP origin, *Plasmodium knowlesi*, is driven by deforestation in Southeast Asia [54]. Similarly, human encroachment into and conversion of NHP habitat may be driving YF spillover from NHP to humans in the Amazon basin.

While the boundary separating high and low NHP reservoir richness regions was based on data, there may be scope for further development of this feature of the model. Specifically, the different seasonality of spillover risk between the regions highlights the need to study individual NHP species’ ability to transmit YFV. This is particularly important given that potential non-human primate reservoirs are present in all but 21 municipalities in Brazil. The NHP species included in the model are from genera known to be susceptible to YF as detected by active infection or antibody titers [3, 16, 55]. However, little is known about individual species’ competencies, which is especially important given the known role of highly competent species in sustaining transmission amongst reservoirs of other vector-borne diseases [56–58]. In addition to competency, data on the relative abundance of NHP populations and mosquitoes’ biting preferences would help identify NHP species most likely to contribute to YF spillover. A better understanding of species’ competencies and ability to transmit YFV to mosquitoes would narrow down the current list of NHP reservoirs to those which genuinely play a role in viral transmissions. This could identify other municipalities without a ‘true’ NHP reservoir present, and therefore not at risk of YF spillover. Future work should address the variation in reservoir competency between species, and the impact of NHP community composition on sylvatic transmission.

Our logistic bagging approach performs well given that the dataset was extremely sparse, with municipalities reporting spillover events approximately 0.01% of the time. The lower testing AUC for the HRR Regional model (AUC = 0.63) is likely due to the sparse dataset. This region only had 20 spillover events during the 13 year period, which was further split into training and testing observations (Table 2). The model performance is based on accurately classifying five presences from the 9926 background points.

Logistic bagging may be useful in other situations where over fitting models to sparse data is a concern, such as St. Louis encephalitis, Lassa fever, or Rift Valley fever. Additionally, a binary response variable can be more robust than a continuous response variable. First, a binary response buffers against gradual improvements in surveillance practices, which is most likely the case for YF in Brazil, since single and multiple cases per an observational unit are equally weighted. This is supported by the lack of an overall increase in risk over the study period. Additional precaution, however, would be needed if a surveillance practices were to dramatically change. For example, this approach would not be suitable to handle case data spanning the implementation of surveillance infrastructure development completed in the early 2000s [59]. Secondly, for the sylvatic YF spillover system, the logistic response variable avoids classifying cases resulting from sylvatic and urban transmission. While the most recent YF outbreaks in Brazil may include cases resulting from both urban and sylvatic transmission, it is doubtful that urban transmission occurred during our study (2001–2013) [5].

This approach is limited by the availability of environmental and epidemiological data that are both continuous and have high temporal and spatial resolution. For neglected diseases, epidemiological data at the sub-administrative level is not always available nor current. Further, remotely sensed environmental data are often at spatial resolutions that are coarser than the disease data, or are averaged over time. For example, daily variation in temperature is shown to have larger effects on mosquito dynamics than mean daily temperature alone [27], but satellite imagery collected daily is unable to measure such daily variation. Processing of satellite imagery is rarely standardized and is not continuous over time due to changes in satellite technology [60], making comparisons across datasets difficult [61]. This limitation is reflected in the current study, whose study period coincides with that of the availability of land cover data, preventing us from predicting YF spillover into the present.

## Conclusions

The spillover risk of YF in Brazil varies in space and time. When spillover risk is modeled using separate models for different ecological contexts (i.e. low *vs* high NHP species richness), differing seasonal patterns emerge. Specifically, when combining predictions across low and high non-human primate richness regions, the asynchrony of spillover seasonality can lead to intermittent hot spots of YF spillover along urban coastal areas, which coincide with 2016/2017 and 2017/2018 YF outbreaks. These seasonal patterns may be due to differences in environmental drivers across a large geographic area and should be further explored through ecological and entomological studies. Understanding these patterns of YF spillover could better inform allocation of public health services and resources, such as vaccination campaigns, which is particularly important given the current shortage in the YF vaccine [62]. Predictive models of disease spillover should incorporate fine-scale temporal and spatial resolutions to better approximate the ecological context and processes driving spillover events.

## Additional files

Additional file 1: **Text 1.** Additional Methods. Methods for data collection, preliminary data exploration, and additional analyses of results. **Table S1.** Results of univariate analyses. Mean AUC values (± SD) of univariate bagged logistic regression models on training dataset using spatial minima, mean, or maxima of rainfall and temperature. **Figure S1.** Correlation matrix of full set of covariates. Numeric inset represents Pearson correlation coefficient. Highly correlated covariates were not included in the models. **Figure S2.** Histogram of number of cases reported in municipality-month. The number of cases reported per municipality-month ranged from 0–18. Municipality-months without cases were not plotted. **Figure S3.** Histogram of municipalities with reoccurring spillover events. **Figure S4.** Long-term trends in predicted YF spillover. Results of slope tests exploring the change in predicted spillover intensity in each municipality across all 156 months. Values represent average change over all 156 months. Non-significant (alpha = 0.05) slopes are reported as zero. **Figure S5.** Long-term trends in predicted YF spillover by calendar month. Results of slope tests exploring the change in predicted spillover intensity in each municipality and month of the year from 2001 to 2013. Values represent average yearly change for each month. Non-significant (alpha = 0.05) slopes are reported as zero. (PDF 3636 kb)

Additional file 2: Predicted spatial risk of yellow fever. Complete time series of predictions. Black dots indicate a municipality reporting any YF cases. (GIF 6840 kb)

## Abbreviations

HRR: High reservoir richness
LRR: Low reservoir richness
NDVI: Normalized Difference Vegetation Index
NHP: Non-human primate
SD: Standard deviation
YF: Yellow fever
YFV: Yellow fever virus
