# Supplementary material for: Spatio-temporal spillover risk of yellow fever in Brazil
Source: Parasit Vectors. 2018 Aug 29;11:488. doi: 10.1186/s13071-018-3063-6 (PMC6116573; doi:10.1186/s13071-018-3063-6)
Supplement: Supplementary file 1 — Text 1. Additional Methods. Methods for data collection, preliminary data exploration, and additional analyses of results. Table S1. Results of univariate analyses. Mean AUC values (± SD) of univariate bagged logistic regression models on training dataset using spatial minima, mean, or maxima of rainfall and temperature. Figure S1. Correlation matrix of full set of covariates. Numeric inset represents Pearson correlation coefficient. Highly correlated covariates were not included in the models. Figure S2. Histogram of number of cases reported in municipality-month. The number of cases reported per municipality-month ranged from 0–18. Municipality-months without cases were not plotted. Figure S3. Histogram of municipalities with reoccurring spillover events. Figure S4. Long-term trends in predicted YF spillover. Results of slope tests exploring the change in predicted spillover intensity in each municipality across all 156 months. Values represent average change over all 156 months. Non-significant (alpha = 0.05) slopes are reported as zero. Figure S5. Long-term trends in predicted YF spillover by calendar month. Results of slope tests exploring the change in predicted spillover intensity in each municipality and month of the year from 2001 to 2013. Values represent average yearly change for each month. Non-significant (alpha = 0.05) slopes are reported as zero. (PDF 3636 kb) [file 13071_2018_3063_MOESM1_ESM.pdf]

# Spatio-temporal Spillover Risk of Yellow Fever in Brazil

RajReni B. Kaul, Michelle V. Evans, Courtney C. Murdock, John M. Drake

## Contents

**1 Data Collection** **2**

1.1 Univariate analysis of covariates . . . 3

**2 Results** **5**

2.1 Summary of YF cases in Brazil . . . 5

2.2 Supplemental results figures . . . 6

# 1 Data Collection

Monthly confirmed cases of yellow fever for each Brazilian Município (sub-state administrative units) Infecção (municipality of infection) from 2001 to 2013 were downloaded from the Brazilian government's portal da saúde website, `tabnet` (`http://tabnet.datasus.gov.br`) on 20 June 2018. Confirmed cases were reported by the Ministry of Health Notification of Injury Information System (SINAN-Net) as determined using clinical-epidemiological criteria.

The annual population for each Município from 2001 to 2013 was also downloaded from the Brazilian government's portal da saúde website,`tabnet` on 05 June 2017. The estimated population was calculated by the Instituto Brasileiro de Geografia e Estatística (Brazialian Institure of Geography and Statistics) as intercensorial estimates.

Monthly land surface temperature and normalized difference vegetation index (NDVI) data from 2001 through 2013 were downloaded from the NASA Land Processes Distributed Active Archive Center (LP-DAAC). The MODIS MOD11C3 product contains monthly temperature data at a $0.05^\circ$ resolution. The MODIS MOD13A3 product contains monthly NDVI data at a 1 km resolution. Both gridded data products were then aggregated to the municipality level to obtain a monthly spatially averaged temperature and NDVI value for each municipality.

Rainfall data was obtained from the NASA GESDISC data archive in the form of data from the Tropical Rainfall Monitoring Mission from 2001 through 2013. The 3B43 product contains an average rainfall rate for each month at a $0.25^\circ$ resolution. We aggregated the gridded data to the municipality level by taking the spatial mean.

Monthly fire locations were downloaded from the Fire Information for Resource Management System (FIRMS). The MODIS Active Fire Product (MCD14ML) reports fire at a 1 km resolution by month. This data was then aggregated to the total number of fires per municipality by month, and scaled to density by dividing by the municipality's total area.

Primate species richness data was obtained from the IUCN Redlist of Terrestrial Mammals for species in genera known to be susceptible to yellow fever (*Ateles, Aotus, Alouatta, Saimiri, Cebus, Callicebus, Callithrix, Saguinus, Lagothrix*) [1, 2]. Individual species' ranges were combined to calculate the number of species found within a municipality, defined as species richness. We also estimated the relative proportion of primate habitat overlapping with agricultural land use per municipality per year. Shapefiles of geographic ranges of each genus were constructed from the above range maps. Yearly land cover data from 2001 - 2013 was downloaded from the NASA Land Processes Distributed Active Archive Center (LPDAAC). The MODIS MCD12Q1 dataset contains yearly land cover categories at a 1 km resolution by year. The proportion of

total municipality area that was both agricultural land use and within a genus range was then calculated for each genus. These proportions were then summed over all nine genera, resulting in a value from 0 - 9 per municipality by year, defined as the Agricultural and Primate Overlap.

Probability of mosquito vector species occurrence was downloaded from the VectorMap Data portal (http://vectormap.si.edu/) on May 30 2018. These are published MaxEnt models of the probability of species occurrences based on environmental covariates and known species presence points. We downloaded data for South and Central America coverage of *Hg. leucocelaenus, Hg. janthinomys, and Sa. chloropterus* (the only species available that are known sylvatic reservoirs of YFV). These models were created in 2011 by the Walter Reed Biosystematics Unit (WRAIR, Division of Entomology). The data is static and at a spatial resolution of 0.04167 °. To calculate the maximum probability of a vector occurrence, we created a spatial average value of vector occurrence for each municipality and species and selected the maximum value of the three species to use for each municipality.

## 1.1 Univariate analysis of covariates

Preliminary data extraction and exploration involved the calculation of the spatial minima, mean, and maxima of temperature and rainfall for each municipality-month. However, these values were extremely correlated with each other (Figure 1). Therefore, we conducted univariate analyses of each variable on the national training dataset using a bagged logistic regression with 50 bags to determine if the spatial mean was an appropriate metric to represent spatial variation in these variables and if it had high explanatory power. When comparing AUC between models (our chosen metric of model performance), the ability to predict on the training dataset was comparable amongst types of spatially aggregated variables (Table 1).

Table 1: Results of univariate analyses. Mean AUC values ($\pm$ *s.d.*) of univariate bagged logistic regression models on training dataset using spatial minima, mean, or maxima of rainfall and temperature.

| | Rainfall | Temperature |
|---|---|---|
| Minimum | $0.631 \pm 0.056$ | $0.530 \pm 0.55$ |
| Mean | $0.643 \pm 0.093$ | $0.501 \pm 0.016$ |
| Maximum | $0.694 \pm 0$ | $0.517 \pm 0.038$ |

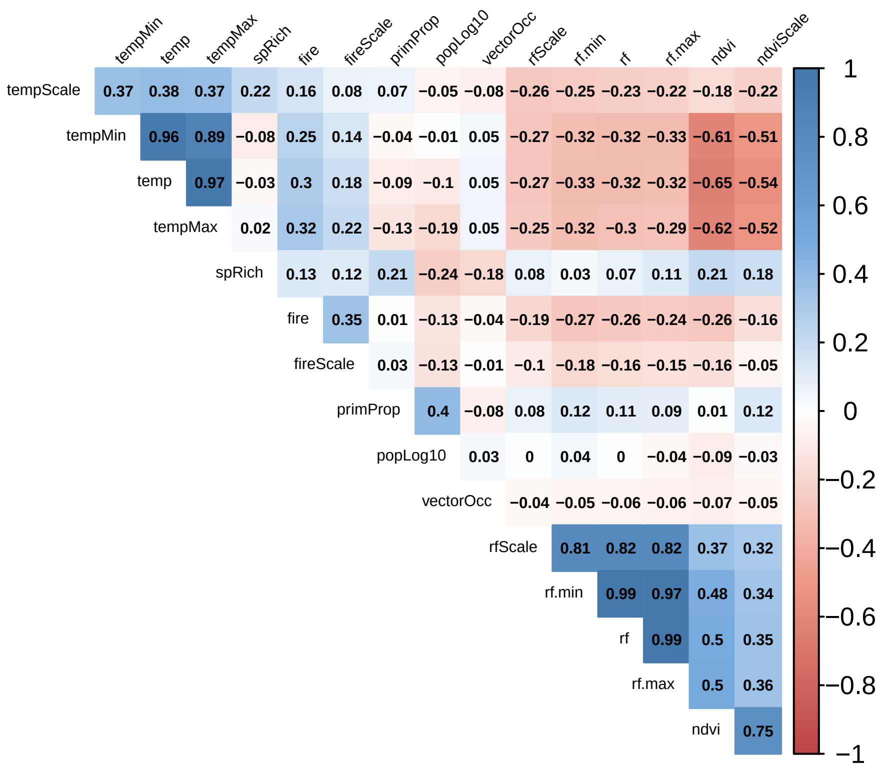


Figure 1: Correlation matrix of full set of covariates. Numeric inset represents Pearson correlation coefficient. Highly correlated covariates were not included in the models.

# 2 Results

## 2.1 Summary of YF cases in Brazil

The majority of municipalities reporting cases, reported a single case in the monthly reporting window (Figure 2). The relatively consistent occurrence of single cases per municipality-month supported the collapse of a continuous case counts into a binary spillover variable. Municipalities rarely reported multiple months with a spillover event (Figure 3). However, when municipalities reported cases in more than two months, they were likely consecutive. Leandro Ferreira, Sabinópolis, and Santo Antônio de Monte reported cases in three months. The latter two municipalities reported cases in consecutive months. Serro, which reported cases in four months, also had three months of consecutive cases. Municipalities with consecutive reporting months also had higher case counts in a single month.

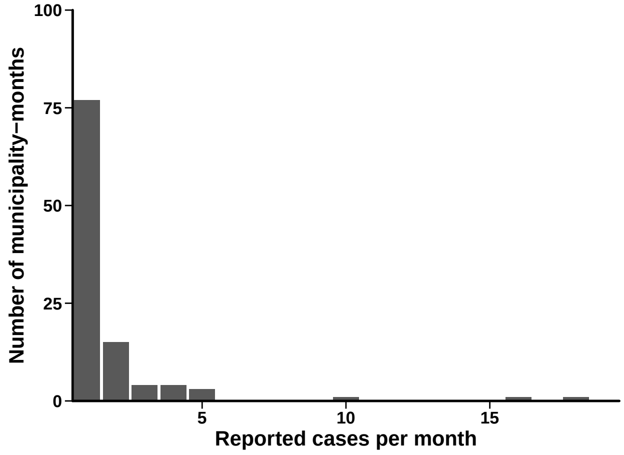


Figure 2: Histogram of number of cases reported in municipality-month. The number of cases reported per municipality-month ranged from 0-18. Municipality-months without cases were not plotted.

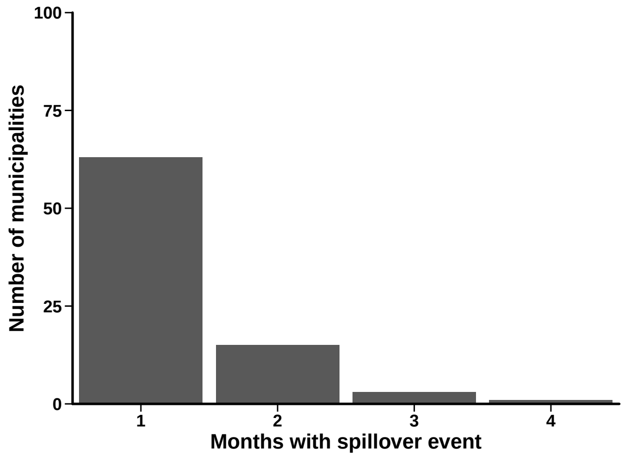


Figure 3: Histogram of municipalities with reoccurring spillover events.

## 2.2 Supplemental results figures

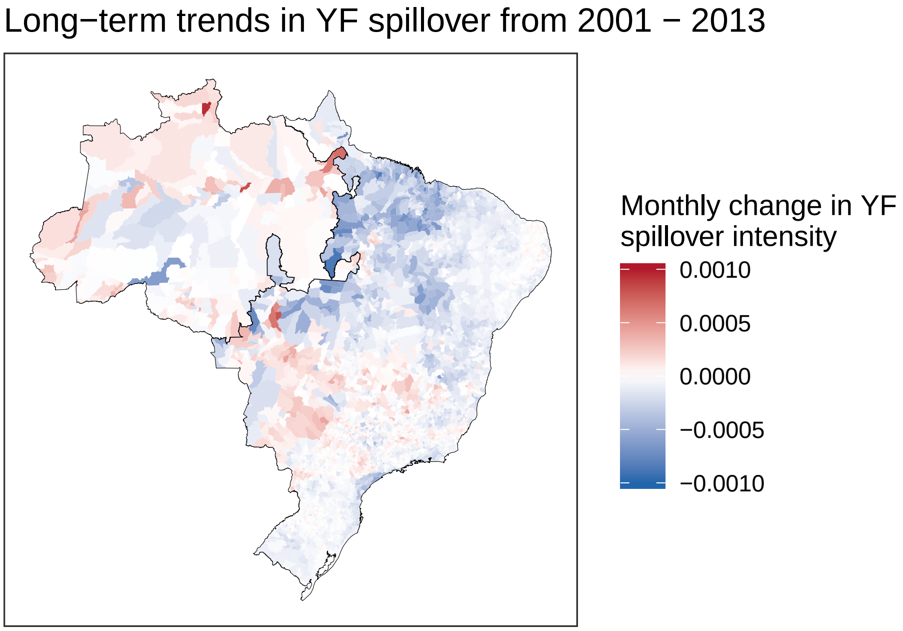


Figure 4: Long-term trends in predicted YF spillover. Results of slope tests exploring the change in predicted spillover intensity in each municipality across all 156 months. Values represent average change over all 156 months. Non-significant (alpha = 0.05) slopes are reported as zero.

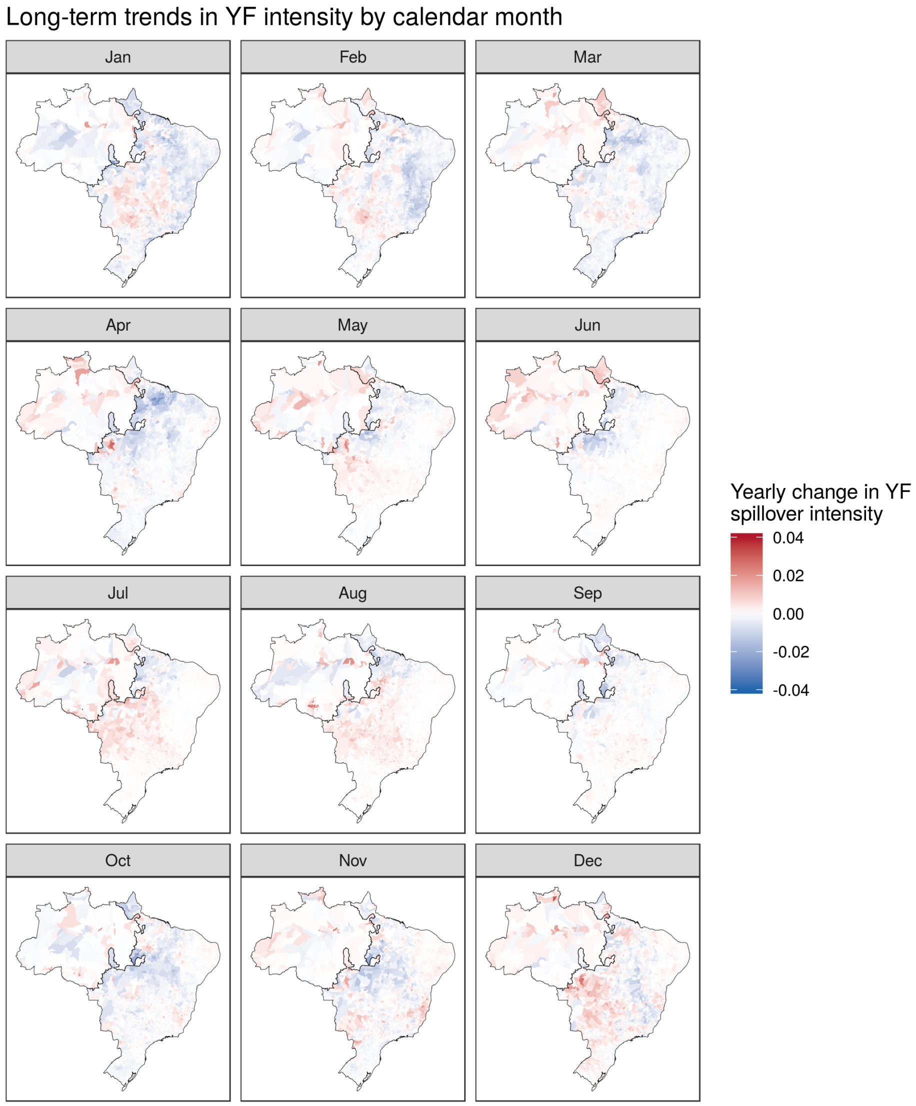


Figure 5: Long-term trends in predicted YF spillover by calendar month. Results of slope tests exploring the change in predicted spillover intensity in each municipality and month of the year from 2001 - 2013. Values represent average yearly change for each month. Non-significant (alpha = 0.05) slopes are reported as zero.

# References

[1] Bicca-Marques, J.C., de Freitas, D.S.: The Role of Monkeys, Mosquitoes, and Humans in the Occurrence of a Yellow Fever Outbreak in a Fragmented Landscape in South Brazil: Protecting Howler Monkeys is a Matter of Public Health. Tropical Conservation Science **3**(1), 78–89 (2010). doi:10.1177/194008291000300107

[2] Hamrick, P.N., Aldighieri, S., Machado, G., Leonel, D.G., Vilca, L.M., Uriona, S., Schneider, M.C.: Geographic patterns and environmental factors associated with human yellow fever presence in the Americas. PLOS Neglected Tropical Diseases **11**(9), 0005897 (2017). doi:10.1371/journal.pntd.0005897
