# Supplementary figures and images for: Spatio-temporal spillover risk of yellow fever in Brazil
Source: Parasit Vectors. 2018 Aug 29;11:488. doi: 10.1186/s13071-018-3063-6 (PMC6116573; doi:10.1186/s13071-018-3063-6)

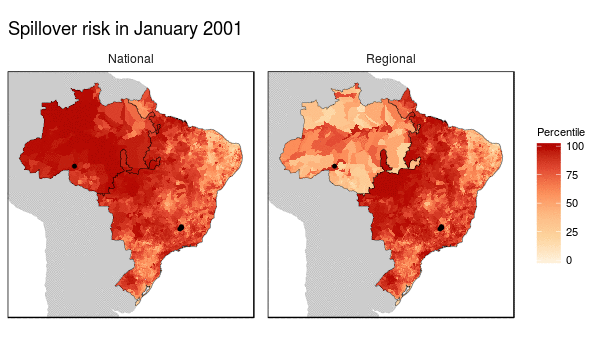

Supplement: Supplementary file 2 — Predicted spatial risk of yellow fever. Complete time series of predictions. Black dots indicate a municipality reporting any YF cases. (GIF 6840 kb) [file 13071_2018_3063_MOESM2_ESM.gif]
